# Dual Tragedy of Fetal and Maternal Loss: A Case of Acute Liver Failure in the Third Trimester

**DOI:** 10.7759/cureus.59421

**Published:** 2024-04-30

**Authors:** Jyotish Guria, Rakesh K Gupta

**Affiliations:** 1 Forensic Medicine and Toxicology, Manipal Tata Medical College, Jamshedpur, IND; 2 Pathology and Laboratory Medicine, All India Institute of Medical Sciences, Raipur, IND

**Keywords:** preeclampsia, liver disease, third trimester, pregnancy, liver failure

## Abstract

The traditional criteria for diagnosing preeclampsia include a new onset of hypertension and new-onset proteinuria at 20 weeks gestation. However recent studies suggest preeclampsia and even eclampsia may develop in the absence of either proteinuria or hypertension. This paper reports a dual tragedy of maternal and fetal loss after 36 weeks in the third trimester. Autopsy findings revealed an enlarged liver with multiple patchy hemorrhages, and histopathology confirmed submassive hepatic necrosis. Early diagnosis with timely referrals to higher centers is always helpful for the patients in such cases.

## Introduction

Pregnancy causes a variety of changes that can impact many organs including the liver. These may range from normal physiologic changes as a result of pregnancy to rapidly progressing serious maternal and fetal outcomes. Such liver function changes can be further divided into those specific and non-specific to pregnancy. Conditions such as preeclampsia and eclampsia, AFLP (acute fatty liver of pregnancy), HELLP syndrome (hemolysis, elevated liver enzymes, and low platelet count), cholestasis, and hyperemesis gravidarum can be allotted as changes specific to pregnancy. Acute liver failure in pregnancy is a rare condition but is associated with a high potential for morbidity and mortality to the mother and fetus [1]. Its causes can be either specific or non-specific to pregnancy. Preeclampsia is a disorder unique to pregnancy presenting usually in the third trimester. The traditional criterion for diagnosing preeclampsia includes a new onset of hypertension and new-onset proteinuria at 20 weeks gestation. However, recent studies suggest preeclampsia and even eclampsia may develop in the absence of either proteinuria or hypertension [2]. Liver involvement in preeclampsia is not common; however, its presence signifies severity [3]. Early and accurate diagnosis of acute liver failure is vital for its management [1,2,4].

## Case presentation

On day one, a 22-year-old primigravidae female with a nine-month pregnancy was brought to a private clinician after complaints of abdominal pain, where she was found to have raised blood pressure (BP), and sublingual nifedipine and magnesium sulfate injection were administered. Later on, she was taken to a government hospital on the same day.

On day two, she was admitted to another private healthcare center. Where on examination, her general condition was noted as poor with a pulse of 156/min and BP of 140/80 min. Blood examinations are shown in Table 1.

**Table 1:** Blood examination results AST, aspartate aminotransferase; ALT, alanine aminotransferase

Test name	Result	Unit	Normal values
Hb	12.6	gm%	13.0-17.0
TLC	8200	cell/cumm	4000-10000
Neutrophils	60%	%	40-80
Lymphocytes	33.1%	%	20-40
Absolute platelet count	2.06	lakhs/mm^3^	1.50-4.10
Total bilirubin	3.72	mg/dL	0.0-<1.0
Bilirubin direct	1.78	mg/dL	0.0-<0.6
Bilirubin indirect	1.94	mg/dL	0.0-1.0
AST	387.99	U/L	15-40
ALT	285.03	U/L	10-40
Albumin	2.67	g/dL	3.5-5.1
Globulin	3.37	g/dL	1.9-3.5

**Figure 1:**
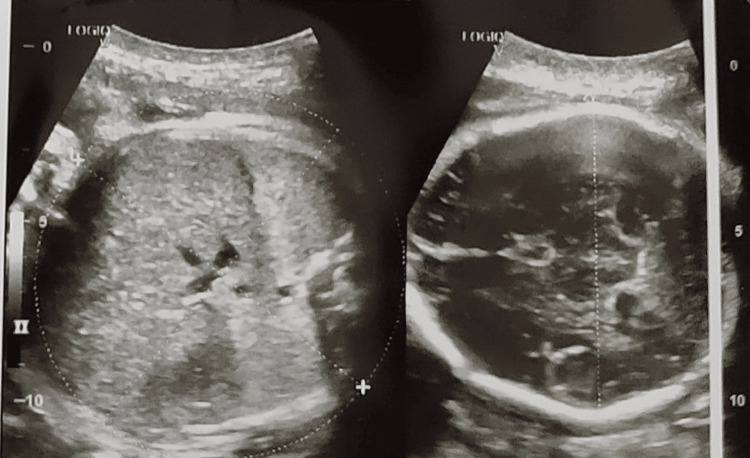
USG of gravid uterus

Fetal heart sound was found to be absent; cervical os was 1.5-2 cm dilated and 20-30% effaced, head high. On USG examination, a single intrauterine fetal demise of 36 weeks two days with the cephalic presentation was diagnosed with absent fetal cardiac activity and absent fetal body and limb movements (Figure 1). She was advised of induction of labor and referred to the tertiary center for ICU management.

She was taken to a higher center in the late night and was declared dead on arrival at around 02.30 am on day three. A medicolegal autopsy was performed to determine the cause of death on the same day afternoon.

Autopsy

External Examination

The female was averagely built with rigor mortis well developed and postmortem lividity present on dorsal and lateral aspects of the body and was fixed. The abdomen was tense with linea nigra in midline extending across the umbilicus. No evidence of external injuries was found. No signs of decomposition were present at the time of examination.

Internal Examination

On opening the skull, the meninges were intact. The brain was pale and edematous. Pleural cavities on each side contained about 500 mL of clear straw-colored fluid. On the opening of the abdominal cavity, the peritoneum was found to be tensed containing about 1500 ml of clear straw-colored fluid in the cavity.

**Figure 2:**
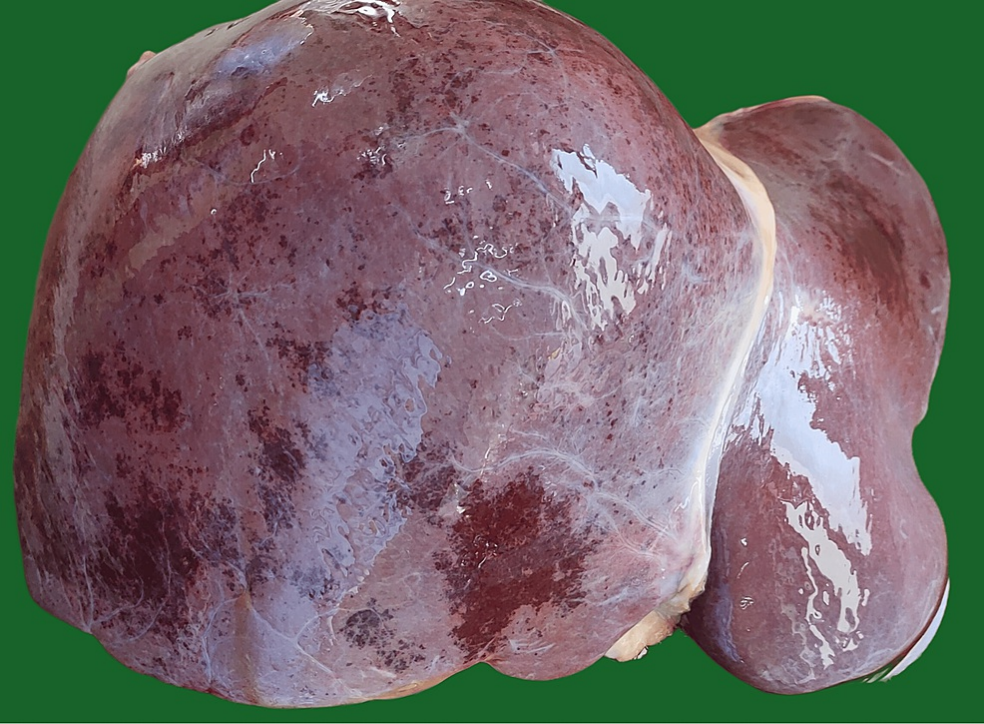
Gross appearance of liver showing multiple hemorrhagic areas

**Figure 3:**
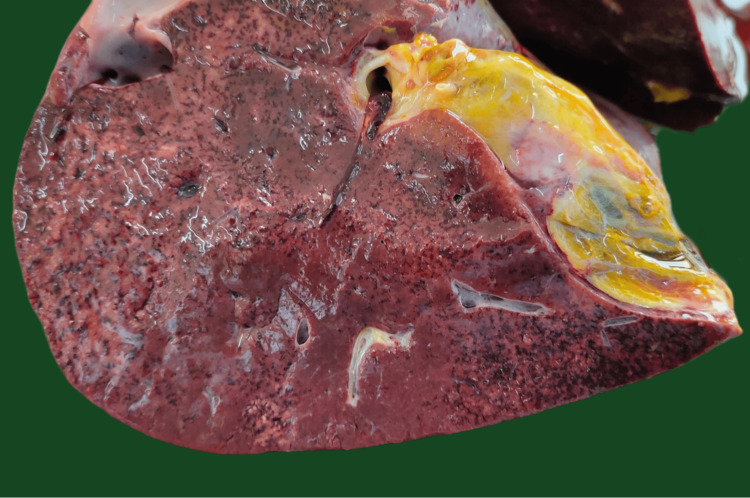
Cut section of the liver showing hemorrhagic areas

No gross cardiac or lung pathology was observed. The liver was enlarged, weighing 1923 gm, with multiple petechial/patchy hemorrhagic areas present over the surface (Figure 2). The cut section of the liver revealed multiple patchy hemorrhages (Figure 3). The uterus was enlarged and a dead male fetus of length 44 cm, weighing 2426 gm, was delivered. Both kidneys were pale with slight blackish discoloration over the surface. Sections of tissues from the lungs, liver, kidneys, brain, and uterus were sent for histopathological examination.

**Figure 4:**
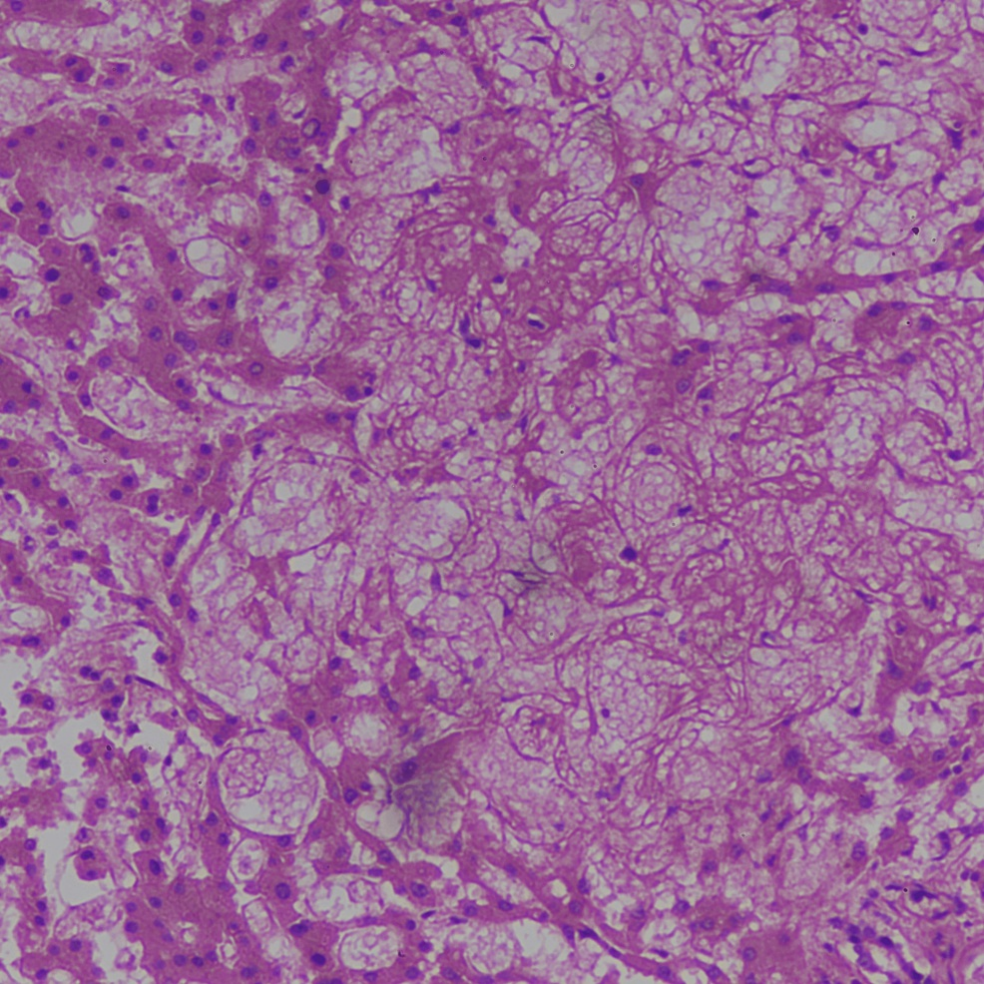
Microscopic examination of the liver showing loss of lobular architecture

On histopathological examination, sections of the liver examined showed loss of lobular architecture (Figure 4). There was marked sinusoidal dilatation, congestion, and focal hemorrhage. Submassive hepatic necrosis is seen. Reticulin stain showed partial collapse of architecture.

Sections from the lungs showed features of pulmonary edema. Section of the uterine wall showed only congestion of blood vessels. Sections from the brain and kidneys were unremarkable. Based on gross autopsy findings and histopathological examination, it was opined that death in this case is due to acute liver failure consequent upon preeclampsia (a natural cause).

## Discussion

Abnormal liver tests together with features of preeclampsia should always raise concern in diagnostic and therapeutic decisions. The presence of both newly onset proteinuria and hypertension at 20 weeks gestation is the conventional criterion for the diagnosis of preeclampsia. However new studies suggest some may develop preeclampsia or eclampsia without having either hypertension or proteinuria [2,5-9]. A study by Sibai et al. suggests an overlapping spectrum of hypertension, capillary leak, maternal symptoms, and hemolysis in atypical preeclampsia [2]. One of the major target organs in preeclampsia is the liver.

There are other conditions that might lead to liver failure during pregnancy; these can be grouped as those specific and non-specific to pregnancy [1].

Preeclampsia and eclampsia, AFLP, and HELLP syndrome are some of the pregnancy-associated conditions leading to acute liver disease. Viral hepatitis, drug-induced hepatitis, toxins, and Budd-Chiari syndrome are some conditions non-specific to pregnancy [4,5,10]. The presence of pregnancy-induced hypertension and ascites favors pregnancy-specific liver diseases [2].

Besides the cause and severity of liver failure, the outcome also depends on early diagnosis and management along with timely referral to the higher centers with better care [1]. The non-specificity of the symptoms always poses a challenge to the physician in the timely diagnosis. Treatment of patients with atypical manifestation includes a careful approach including maternal risk, clinical, lab, and imaging results.

A team approach is necessary since the outcome may rapidly escalate toward substantial morbidity for both the mother and the fetus. A multidisciplinary strategy involving obstetricians and gastroenterologists/hepatologists is necessary for the management of liver disease in pregnancy [11-13]. Liver transplantation is the only viable option when acute liver failure is not likely to recover [2,3].

In a study by Harmon et al., it was suggested that clinically apparent preeclampsia is associated with a high risk of fetal death [14]. Expedited delivery of the fetus with optimal maturity is the mainstay of the treatment in conditions leading to liver failure in pregnancy [1].

## Conclusions

This case report emphasizes the importance of early identification and treatment together with prompt referral for preeclamptic patients. Non-specificity of the symptoms makes the diagnosis difficult for the physician. Preeclampsia may lead to fatal consequences even in the absence of defining features. Thus, a high index of suspicion should be kept for pregnant patients presenting with hypertension and deranged liver functions. Early diagnosis can help the patient with timely referrals to higher centers. Also in cases of sudden maternal deaths, a complete clinical history and the knowledge of the disease progression is necessary to come to the final opinion.
